# A 10-year follow-up service evaluation of the treatment pathway outcomes for patients in nine in-patient psychiatric rehabilitation services

**DOI:** 10.1192/bjb.2021.123

**Published:** 2023-02

**Authors:** Tom Edwards, Alan Meaden, Martin Commander

**Affiliations:** 1 Gloucestershire Health and Care NHS Foundation Trust, UK; 2 Birmingham and Solihull Foundation Trust, UK

**Keywords:** Rehabilitation, schizophrenia, psychotic disorders, outcome studies, in-patients

## Abstract

**Aims and method:**

This study examines the treatment pathway outcomes over a 10-year period for patients in nine rehabilitation wards at the beginning of this time period.

**Results:**

Data were obtained on 85 patients, of whom 59 were discharged during the 10-year period; 29 were readmitted, of whom 15 had further in-patient rehabilitation admissions. Nineteen patients remained in hospital throughout the period. Only nine patients were living independently at the time of follow-up or death, and 34 were in longer-term in-patient settings. Eighteen patients had died during the 10-year period.

**Clinical implications:**

New planning of rehabilitation services needs to ensure an integrated whole-systems approach, across in-patient and community settings, with specialist mental health rehabilitation teams to support people moving from hospital to the community, and for the small number remaining in hospital for very long periods, development of sufficient high-quality, local in-patient provision.

Mental health rehabilitation services work with low-volume, high-need individuals, with long-term complex mental health and social needs.^1^ Despite the introduction of intensive community services such as assertive outreach teams, there remain a small but significant group of patients who require specialist in-patient provision over a sustained period.^2^ Rehabilitation psychiatry remains an area of clinical practice that has been described as an evidence-free zone in modern psychiatry.^3^ There are a limited number of descriptions of in-patient rehabilitation services and remarkably little information on patient pathway outcomes. The data available to date indicate that around two-thirds of people supported by rehabilitation services progress to successful community living within 1.5 years of admission to an inpatient rehabilitation unit. Furthermore, two-thirds of patients maintain this progress over 5 years, without requiring a further hospital stay, and one in ten achieve independent living within this period.^4,5^

This study expands on the knowledge of rehabilitation in-patient services, by investigating various treatment pathway outcome measures for patients from a previous study undertaken in 2007 by Cowan et al.^6^ This service evaluation aimed to further an understanding of the evidence for the need for rehabilitation in-patient services, with reference to the currently limited research into the longer-term complexities of patients’ rehabilitation pathways, when taken over a long period of time.

## Method

The original study was a descriptive survey that was determined by the national research ethics service to be a service evaluation, for which ethical committee approval was not required. Approval was gained from the Research and Development department at Birmingham and Solihull Mental Health Foundation Trust, who classified this follow-up study as being a service evaluation in accordance with Health Research Authority guidance.^7^ This study was designed to describe the care pathways provided for these patients over the 10 years since the original study. It reports measures utilised by current services without reference to any standard set of outcomes.

### Sample

The sample from the original study comprised patients in ten in-patient rehabilitation wards at the time of an initial descriptive survey undertaken between March and June 2007.^6,8^ These wards, provided by two National Health Service (NHS) Trusts, were defined as NHS managed and staffed in-patient units, with a stated aim of rehabilitation and providing 24 hour care from mental health nurses and a dedicated consultant psychiatrist. The rehabilitation in-patient units were categorised according to the Royal College of Psychiatrists guidelines in 2009,^9^ as community rehabilitation units (CRUs), longer-term complex care units (CCUs) and high-dependency rehabilitation units (HDUs).

### Data

The follow-up data were obtained during July 2017, and predominantly comprised items routinely recorded on clinical information systems. These outcome measures included overall length of stay, placement on discharge from hospital or at the time of death, and the number of patients who were readmitted and had further periods of in-patient rehabilitation over the time period of the study. The data also included community follow-up arrangements for this group of patients over the 10-year period.

There was no direct contact with patients or staff. All data was anonymised and stored in accordance with the protected information technology governance arrangements by Birmingham and Solihull Mental Health Foundation Trust. The patient record was stored separately to the main data, alongside a unique research number to allow cross linkage. The data are presented descriptively.

## Results

### Follow-up sample

Of the 98 patients (out of 109) with available data in the initial survey, 65 were male and the mean age was 45 years. In terms of ethnicity, 66 patients were White, 22 patients were Black or Black mixed, and ten patients were in other groups. None of the patients were employed and only five were married. Sixty-six patients had a diagnosis of schizophrenia, 26 patients had affective psychoses, two patients had a personality disorder, one patient had organic psychosis, one patient had substance misuse disorder and one patient had a diagnosis of ‘other’. A total of 75 patients had come from other psychiatric wards, most commonly acute in-patient wards (for more details, see Cowan et al^6^ and Wolfson et al^9^). One CRU, with ten patients, from the original survey was necessarily excluded. It was provided by a different NHS Trust to the other units and, for several reasons, it proved unfeasible to collect the follow-up data.

Of the remaining eligible patients, 88 (out of 99) had data from the initial survey, and of these, follow-up data was obtained on 85; information on three patients, all from CRUs, was missing. The final sample consisted of 28 patients from four CRUs, 30 patients from three CCUs and 27 patients from two HDUs.

Of the 85 patients, 18 (21%) were deceased at the time of the follow-up (six from CRUs, ten from CCUs and two from HDUs); 12 died in hospital (four from CRUs, six from CCUs and two from HDUs) and the remaining six (two from CRUs and four from CCUs) died in community placements following their discharge (see Table 1). There were no instances of suicide, but other causes of death were not consistently or clearly specified in the records available; the mean age of death was 62 years (s.d. 13 years, range 31–87 years). Of the 22 patients alive and in hospital after 10 years, eight remained in units run by the same NHS Trust, six were in rehabilitation wards run by other organisations but still within the same catchment area, and eight were now in units located elsewhere in England.

**Table 1:** Overall outcome

	Total, *N* = 85		Community rehabilitation unit, *n* = 28		Rehabilitation high-dependency unit, *n* = 27		Longer-term complex care unit, *n* = 30	
	*n*	%	*n*	%	*n*	%	*n*	%
Deceased	18	21	6	21	2	7	10	33
Secure ward^a^	6	26	0	11	6	52	0	17
Rehabilitation ward	16		3		8		5	
Nursing home	10	33	3	36	2	22	5	40
Care home	18		7		4		7	
Supported^b^	9	20	3	32	3	19	3	10
Independent	8		6		2		0	

a. Including low and medium secure units.

b. Shared supported accommodation, including group homes and hostels.

Only around one in ten patients were living in independent accommodation at the time of follow-up or their death, whereas two-fifths were in longer-term in-patient settings (see Table 2).

**Table 2:** Placement at follow-up or time of death

	Total, *N* = 85		Community rehabilitation unit, *n* = 28		Rehabilitation high-dependency unit, *n* = 27		Longer-term complex care unit, *n* = 30	
	*n*	%	*n*	%	*n*	%	*n*	%
Secure ward^a^	6	40	0	25	6	59	0	37
Rehabilitation ward	28		7		10		11	
Nursing home	11	39	3	39	2	22	6	53
Care home	22		8		4		10	
Supported^b^	9	21	3	36	3	19	3	10
Independent	9		7		2		0	

a. Including low and medium secure units.

b. Shared supported accommodation, including group homes and hostels.

### Movement between rehabilitation units

Fifteen patients moved to another rehabilitation unit during their index episode. Three CRU patients moved to a CCU, whereas one CCU patient moved to a ward specifically designated for infirmed older patients and another spent a period on a HRU before returning to the same complex care setting. Most movement occurred within HRUs: of the ten patients who changed ward, five moved to low or medium secure facilities, four moved to CCUs and one moved to a CRU (following a period of time on a low secure ward).

### Discharge from hospital and length of stay

A total of 19 patients remained in hospital throughout the 10-year follow-up period, most of these on an HDU (three on CRUs, three on CCUs and 13 on HDUs). A further eight patients died during their index admission (two from CRUs, five from CCUs and one from an HDU). Nevertheless, 59 (69%) patients were discharged during the 10-year follow-up period. This was higher for patients on CRUs (*n*=23, 82%) and CCUs (*n*=22, 73%) compared with those on HDUs (*n*=14, 52%). There was marked variation in lengths of stay for patients, even within the rehabilitation unit types. The median length of stay for those discharged was 6 years and 10 months (range 5–239 months): 3 years (5–134 months) for patients on CRUs, 6 years and 8 months (10–183 months) for patients on CCUs and 9 years and 7 months (33–239 months) for patients on HDUs.

### Readmission and further episodes of in-patient rehabilitation

Of those discharged, 29 patients (49%) were readmitted; 13 (57%) patients from CRUs, nine (41%) patients from CCUs and seven (50%) patients from HDUs had a further hospital stay. For 11 patients, this was only once, but for the remainder, this ranged from two to eight times. Regarding the 29 patients who were readmitted, 15 (52%) had further periods of in-patient rehabilitation. Of the six patients discharged from CCUs, three were subsequently readmitted to CCUs and three were readmitted to CRUs. Of the five patients discharged from CRUs, two were admitted to CCUs, two were admitted to CRUs (one patient was admitted twice) and one had an especially complicated pathway returning initially to a CRU before then moving to a CCU, and finally an HDU. With reference to the four patients from HDUs, two were readmitted to CRUs, one to a CCU and one to a low secure ward.

### Community follow-up

Of the 67 patients who were alive at the time of follow-up, 36 (54%) were under a community mental health team, 18 (27%) were under an assertive outreach team and 13 (19%) had no follow-up (four of whom were in placements outside the city). The distribution was similar when only considering the 45 patients living in the community at follow-up; 23 (51%) were under a community mental health team, 11 (24%) were under an assertive outreach team and 11 (24%) had no follow-up (two of whom were in nursing homes outside of Birmingham).

## Discussion

A restricted data-set encompassing a small number of units and patients, all from one NHS Trust with a predominantly urban catchment area, inevitably limits the generalisability of the findings from this study.

The results offer some support to the Joint Commissioning Panel for Mental Health's^5^ updated guidance on the typology of rehabilitation wards, with a newly introduced division of HDUs (previously for between 1 and 3 years) into long-term high-dependency (high-support) units (up to 5 years) and high-dependency (high-support) in-patient rehabilitation units (up to 1 year). Furthermore, the Joint Commissioning Panel for Mental Health's advice^5^ on CRUs has increased the length of stay from 1 year to 1–2 years, as well as that for CCUs, from ‘several years’ to a more exacting but perhaps longer period of 5–10 years. In addition, where a local rehabilitation pathway provides either a high dependency (high support) in-patient rehabilitation unit or a community rehabilitation unit (rather than both), the expected length of stay may be up to 3 years.

Nevertheless, the data from this study suggest that the lengths of stay proposed for each type of unit possibly underestimates the time patients currently spend on rehabilitation wards. Two-fifths of patients were in hospital at the follow-up or the time of their death, and the median length of stay for those who were discharged from the HDUs was almost 10 years; this is in accordance with the cohort of very-long-stay psychiatric in-patients highlighted in the Centre for Mental Health briefing on rehabilitation,^10^ but seemingly at odds with the most recent guidance on the various types of rehabilitation wards by the Royal College of Psychiatrists,^11^ which no longer includes CCUs.

Indeed, the recent National Institute for Health and Care Excellence guideline on ‘Rehabilitation for Adults with Complex Psychosis’^12^ draws attention to the need for more research on people who spend very long periods of time in longer-term rehabilitation services. It may well be clinically appropriate for a minority of patients to remain in hospital for many years, but it is necessary to then ask whether units accommodating them, especially where locked and restricting access to the community, have a comprehensive range of psychosocial interventions, including appropriate psychological therapies and meaningful activities, which can enhance patients’ lives even within the confines of their setting.

The Care Quality Commission^13^ has reported the highly variable length of admissions of patients on rehabilitation wards, and expressed concern that some patients, particularly those in poorly defined ‘locked rehabilitation’ units,^14^ may be staying longer than necessary. The picture presented here, with widely differing durations of stay even within the same category of facility, reinforces this impression. Any analysis of the reasons for this must include a searching appraisal of the effectiveness of the interventions provided in these units. Definitions of rehabilitation services typically stress the importance of developing life skills, which help lead to greater independence and are critical to successful community living, with appropriate support.^9^ Yet the evidence from the present study indicates that this may not be a realistic goal for many patients. Similar to the findings of a previous follow-up cohort,^4^ only around one in ten patients progressed to living independently. Occupational therapy, which seeks to improve everyday living skills and engage people in valued activities, has traditionally been at the core of rehabilitation practice. However, a recent major study of in-patient rehabilitation did not detect any clinical advantage of a newly designed activity-based intervention over standard care.^15^ As the authors acknowledge, the patients may have been too severely impaired to be able to sufficiently benefit; an evaluation of innovative new approaches is urgently required.^8,16^

One treatment domain clearly requiring improvement is the standard of physical healthcare in rehabilitation units; one in five patients had died by the time of follow-up, at a mean age of only 62 years. Comorbid physical health problems are common in this patient population and, as has been highlighted in a strategic review of mental health services,^17^ special attention is now crucial to tackle this premature mortality, involving more effective liaison with both primary and secondary care colleagues; the findings here give further weight to this assertion.

Recent commissioning guidance emphasises the importance of a system of rehabilitation services and cohesive rehabilitation care pathways.^5^ In this study, half of the patients who were discharged had a further hospital admission, with a quarter having had more than one episode of care on a rehabilitation unit. It is also notable that nearly two-thirds of the patients in hospital at follow-up were no longer in facilities run by the same NHS Trust, and just over one third were in units outside their original catchment area. The complex pathways of patients, even within in-patient rehabilitation services provided by just one NHS Trust, highlights the importance of effectively integrating a range of types of units while also being able to offer ready access to acute hospital admission. Certainly, the findings in this study contradict the notion of a simple linear flow of patients along a pathway from high-intensity settings through to the community setting and independence. Indeed, most of the patients living outside of hospital at follow-up were in some form of supported accommodation, pointing to the necessity for an approach to the commissioning and provision of services that embraces cooperation across the full gamut of residential options.^18^

Alongside the increasing plurality of in-patient rehabilitation and intensive community support services, the erosion of specialist community rehabilitation teams has arguably contributed to disruptions in patients’ rehabilitation pathways. There were no dedicated community rehabilitation teams in the NHS Trust involved in this study. Although a quarter of the patients were under an assertive outreach team, half were with a community mental health team, and a quarter had no community team involved in their care planning. This is consistent with the national picture, with only one in four NHS Trusts having specialty community rehabilitation services.^19^ It is recognised that the absence of dedicated teams compromises the ability of patients to progress from in-patient to community-based rehabilitation settings and, in turn, sustain these placements over the longer term. The development of these teams is highlighted as a priority in a recent report by Rethink Mental Illness and the Royal College of Psychiatrists.^19,20^

## About the authors

**Tom Edwards** is a Consultant Psychiatrist for the Gloucestershire Rehabilitation Inpatient Service, Gloucestershire Health and Care NHS Foundation Trust, UK. **Alan Meaden** is a Consultant Clinical Psychologist for Integrated Community Care and Recovery Services at Birmingham and Solihull Mental Health NHS Foundation Trust, UK . **Martin Commander** is a Consultant Psychiatrist in Rehabilitation services at Birmingham and Solihull Mental Health NHS Foundation Trust, UK.

## Data availability

The data has not been made available.

## References

[1] Killaspy H. The ongoing need for local services for people with complex mental health problems. Psychiatr Bull 2014; 38: 257–9. 10.1192/pb.bp.114.048470 PMC4248159 25505623

[2] Rana T, Commander M. A long term follow up of patients on assertive outreach teams. Psychiatrist 2010; 34(3): 88–91.

[3] Killaspy H, Harden C, Holloway F, King M. What do mental health rehabilitation units do and what are they for? A national survey in England. J Ment Health 2005; 14: 157–65.

[4] Killaspy H, Zis P. Predictors of outcomes of mental health rehabilitation services: a five year retrospective cohort study in inner London, UK. Soc Psychiatry Psychiatr Epidemiol 2012; 48(6): 1005–12. 22945367 10.1007/s00127-012-0576-8

[5] Joint Commissioning Panel for Mental Health. Guidance for Commissioners of Rehabilitation Services for People with Complex Mental Health Needs. Joint Commissioning Panel for Mental Health, 2016 (https://www.rcpsych.ac.uk/docs/default-source/members/faculties/rehabilitation-and-social-psychiatry/rehab-social---joint-commissioning-panel---guidance-for-commissioners-of-rehabilitation-services-for-people-with-complex-mental-health-needs---2016.pdf?sfvrsn=82cbb5e4_6).

[6] Cowan C, Meaden A, Commander M, Edwards T. In-patient psychiatric rehabilitation services: survey of service users in three metropolitan boroughs. Psychiatrist 2012; 36: 85–9.

[7] NHS Health Research Authority. Defining Research. NHS, 2017 (https://www.clahrc-eoe.nihr.ac.uk/wp-content/uploads/2014/04/defining-research.pdf).

[8] Meaden A, Commander M, Edwards T, Cowan C. Patient engagement and problematic behaviours in residential rehabilitation units. Psychiatr Bull 2014; 38: 260–4. 10.1192/pb.bp.113.045252 PMC4248160 25505624

[9] Wolfson P, Holloway F, Killaspy H. Enabling Recovery for People with Complex Mental Health Needs: A Template for Rehabilitation Services. Royal College of Psychiatrists, 2009 (www.rcpsych.ac.uk/docs/default-source/members/faculties/rehabilitation-and-social-psychiatry/rehab-enabling-recovery-for-people-with-complex-mental-health-needs.pdf?sfvrsn=6b9Of31_4).

[10] Wright E. Briefing 51: Long Stay Rehabilitation Services. Centre for Mental Health, 2017 (www.centreformentalhealth.org.uk/publications/briefing-51-long-stay-rehabilitation-services).

[11] Royal College of Psychiatrists. *Mental Health Inpatient Rehabilitation Services Typology Table*. Royal College of Psychiatrists, 2019 (www.rcpsych.ac.uk/docs/default-source/members/faculties/rehabilitation-and-social-psychiatry/mental-health-inpatient-rehabilitation-services-typology-table-20-3-19.pdf?sfvrsn+8fc19480_4).

[12] National Institute for Health and Care Excellence (NICE). Rehabilitation for adults with complex psychosis NICE Guideline [NG181]. NICE, 2020 (www.nice.org.uk/guidance/ng181). 32991081

[13] Care Quality Commission. The State of Care in Mental Health Services: 2014–17. Care Quality Commission, 2017 (www.cqc.org.uk/publications/major-report/state-care-mental-health-services-2014-2017).

[14] Dye S, Smyth L, Pereira S. Locked rehabilitation: a need for clarification. BJPsych Bull 2016; 40: 1–4. 26958351 10.1192/pb.bp.114.049726 PMC4768839

[15] Killaspy H, Marston L, Green N, Harrison I, Lean M, Cook S, Clinical effectiveness of a staff training intervention in mental health inpatient rehabilitation units designed to increase patients’ engagement in activities (the Rehabilitation Effectiveness for Activities for Life [REAL] study): single-blind, cluster-randomised controlled trial. Lancet Psychiatry 2015; 2: 38–48. 26359611 10.1016/S2215-0366(14)00050-9

[16] Leff J, Szmidla A. Evaluation of a special rehabilitation programme for patients who are difficult to place. Soc Psychiatry Psychiatr Epidemiol 2002; 37: 532–6. 12395143 10.1007/s00127-002-0578-z

[17] Mental Health Taskforce. The Five Year Forward View for Mental Health. NHS England, 2016 (www.england.nhs.uk/wp-content/uploads/2016/fyfv-mh.pdf).

[18] Edwards T, Macpherson R, Commander M, Meaden A, Kalidindi S. Services for people with complex psychosis: towards a new understanding. BJPsych Bull 2016; 40(3): 156–61. 27280038 10.1192/pb.bp.114.050278 PMC4887735

[19] Rethink Mental Illness, Royal College of Psychiatrists. *In Sight and in Mind: Making Good on the Promise of Mental Health Rehabilitation.* Rethink Mental Illness and the Royal College of Psychiatrists, 2020 (www.rethink.org/media/3571/insightandinmind_rehabreport_rethinkmentalillness_rcpsych_february_2020.pdf).

[20] Kalidindi S, Killaspy H, Edwards T. Community Psychosis Services: The Role of Community Mental Health Teams (Faculty Report FR/RS/7). Royal College of Psychiatrists, 2012 (www.rcpsych.ac.uk/docs/default-source/members/faculties/rehabilitation-and-social-psychiatry/rehab-fr-rs-07.pdf).

