# A 10-year follow-up service evaluation of the treatment pathway outcomes for patients in nine in-patient psychiatric rehabilitation services – ERRATUM

**DOI:** 10.1192/bjb.2022.8

**Published:** 2023-02

**Authors:** Tom Edwards, Alan Meaden, Martin Commander

**Keywords:** Rehabilitation, schizophrenia, psychotic disorders, outcome studies, in-patients

The first author's name was incorrectly published in the above article. This has since been corrected. The Publisher apologises for the error.
