# A 10-year follow-up service evaluation of the treatment pathway outcomes for patients in nine in-patient psychiatric rehabilitation services – CORRIGENDUM

**DOI:** 10.1192/bjb.2022.97

**Published:** 2023-02

**Authors:** Tom Edwards, Alan Meaden, Martin Commander

**Keywords:** Rehabilitation, schizophrenia, psychotic disorders, outcome studies, in-patients, corrigendum

This notice is to correct an erroneous reference in the aforementioned paper. In the last paragraph of p. 2 of the paper, there is an erroneous mention of “HRUs”. This should be “HDUs” and the paragraph should read as follows:

Three CRU patients moved to a CCU, whereas one CCU patient moved to a ward specifically designated for infirmed older patients and another spent a period on a HDU before returning to the same complex care setting. Most movement occurred within HDUs: of the ten patients who changed ward, five moved to low or medium secure facilities, four moved to CCUs and one moved to a CRU (following a period of time on a low secure ward).
